# The role of a clinical nurse consultant in an Australian Health District: a quantitative survey

**DOI:** 10.1186/s12912-015-0075-9

**Published:** 2015-05-06

**Authors:** Lesley Wilkes, Lauretta Luck, Jennifer O’Baugh

**Affiliations:** School of Nursing and Midwifery, University of Western Sydney, Locked Bag 1797, Penrith South DC, Sydney, NSW 2751 Australia; Nepean Blue Mountains Local Health District, Penrith, Sydney, NSW 2751 Australia; Centre for Nursing Research and Practice Development, Nepean Hospital, PO Box 63, Penrith, Sydney, NSW 2751 Australia

**Keywords:** Clinical nurse consultant, Advance practice nurse, Domains of practice, Nursing roles

## Abstract

**Background:**

This study replicates previous research undertaken in 2013 that explored the role of the Clinical Nurse Consultant in a metropolitan health district in Sydney, Australia.

**Methods:**

A descriptive survey, using Likert scales, was used to collect data from Clinical Nurse Consultants.

**Results:**

Clinical Nurse Consultants are well informed about the domains and functions of their role, as stipulated in the relevant award. They identified clinical service and consultancy as the area in which they predominantly practice.

**Conclusion:**

Despite the clarity of the domains and functions as outlined in the relevant legislated award, the activities undertaken by these clinical nurses are institutionally, individually and contextually constructed.

## Background

Over the past two decades a number of studies have been published in Australia where much of the work has concentrated on the role of the Clinical Nurse Consultant (CNC) in New South Wales (NSW) and equivalent roles in the health systems nationally [1,2]. While the role of the CNC has been investigated by many there is still no agreement as to what the major functions of the role of these nurses should be. This paper reports the findings of a study which replicated previous research on the role of the CNC in a health service in Sydney, Australia in 2003 [3,4]. This paper will report on the details of the domains and functions that a group of CNCs, in the same health district as the prior study in 2013, considered essential.

The CNC role was established in 1992 in NSW [3,5]. It is seen as equivalent in function to other advanced clinical nursing positions for example Clinical Nurse Specialist which is common in some states of Australia as well as in the United Kingdom (UK), United States of America (USA) and Canada [6-8]. Since 1992, the CNC role in NSW, Australia has been delineated into domains of practice with functions attached to each domain. This is consistent for the three CNC grades used in salary determinations in NSW. Since the original position description for the three CNC grades, in 2011 the domains were revised and there were minor editorial changes [5]. The current five domains are: clinical service and consultancy; clinical leadership; research; education; and clinical service planning [5].

Bloomer and Cross [9], found that the CNC role was diverse and complex, with an underutilisation of these nurses as leaders. They found that organisational consensus on the scope and purpose of the role had not been actualised and that there were few support systems for CNCs. This finding is similar to the results of O’Baugh, Wilkes, Vaughan [3]. Australia moved to national nursing and midwifery registration in 2010 and Duffield, Gardner, Chang [10] called for national consistency in nomenclature as the titles seemed to be governed by industry rather than the profession. This group has researched the role of advanced practice nurses during the past decade and have developed a tool [2,11,12] to investigate the CNC role which has similar but slightly different descriptors of the functions/activities than those standardised in NSW [5]. The five domains of activities defined by this research team are: clinical service and consultancy, clinical leadership, research, education and clinical service planning and management. Each of their domains has ten hierarchical categories reflecting complexity and involvement. Fry, Duffield, Baldwin [2] provide another schema and defined four overall title descriptors of the CNC role. These are:

- Sole practitioner: has deep technical expertise in a narrow area of clinical speciality, may be informally part of a multidisciplinary team but works primarily independently with a few patients/clients across a number of settings.
- Clinical co-ordinator: has deep technical expertise in a narrow area of clinical speciality, key member of a multidisciplinary team providing regular outpatient clinics to many patients/clients, not across settings but in a care management role within a clinic.
- Team co-ordinator: has deep technical expertise in a narrow area of clinical speciality is a key member of a multidisciplinary team providing direct clinical care to many inpatients, may be a number of wards and clinics.
- Clinical leader has a high degree of technical expertise in a broad range of specialities, works as an independent practitioner focusing primarily on clinical leadership and research with a few patients/clients [2].

The aim of the current paper is to report on the importance and prioritisation of the CNC domains and functions as seen by the nurses in one health district in Sydney, NSW, Australia in 2013.

## Methods

A quantitative design was used in this study. Data were collected using a survey instrument. The items were close-ended and measured on Likert scales. Likert scales was the most appropriate method to collect the CNC’s opinions about their roles [13]. The participants were asked to rank the domains and functions of their role. First, they were asked to rank how much a particular domain plays a part in their role and then to rank how much a domain should play a part in their role.

### Setting and participants

Fifty-two CNCs and three CMCs (Clinical Midwife Consultants) employed in an Australian metropolitan health district as of 1^st^ January 2013 were sent an invitation.

### Survey tool

The survey was developed using the template of the original 2003 study [3]. Data were uploaded into SPSS [14]. The survey consisted of five parts:

1. Demographics related to age, gender, clinical experience, speciality experience and education.
2. Domains and functions of role from NSW Health [7] were listed and respondents asked to estimate how many hours per month they worked in this domain.
3. Respondents were asked how often each domain played, and how often it should play, a part in their role using the scale of *not at all, occasionally, frequently*.
4. Respondents were asked to tick each of the functions related to each domain that described their work in that domain.
5. Respondents were asked to classify themselves according to the role descriptors of sole practitioner, clinical co-ordinator, team co-ordinator clinical leader developed by Fry, Duffield, Baldwin [2]

Detail about the domains and functions of the CNC roles, as articulated in the NSW Health Award [5], are presented in Tables 1, 2, 3, 4, 5 and 6.

**Table 1:** **CNCs perceptions of how important each domain of practice is in their current position and how important it should be (n = 16)**

	**Plays a part in my CNC role**			**Should play a part in my CNC role**		
**Domains**	**Grade 1**	**Grade 2**	**Grade 3**	**Grade 1**	**Grade 2**	**Grade 3**
	***n*** **= 6 (%)**	***n*** **= 9 (%)**	***n*** **= 1 (%)**	***n*** **= 6 (%)**	***n*** **= 9 (%)**	***n*** **= 1 (%)**
Clinical Services Planning & Management						
Not at all	0 (0)	0 (0)	0 (0)	0 (0)	0 (0)	0 (0)
Occasionally	2 (33)	4 (44)	1 (100)	1 (17)	0 (0)	1 (100)
Frequently	4 (67)	5 (56)	0 (0)	5 (83)	9 (100)	0 (0)
Clinical Services & Consultancy						
Not at all	0 (0)	0 (0)	0 (0)	0 (0)	0 (0)	0 (0)
Occasionally	1 (17)	1 (11)	1 (100)	1 (17)	0 (0)	1 (100)
Frequently	5 (83)	8 (89)	0 (0)	5 (83)	9 (100)	0 (0)
Clinical Leadership						
Not at all	0 (0)	0 (0)	0 (0)	0 (0)	0 (0)	0 (0)
Occasionally	1 (17)	0 (0)	1 (100)	1 (17)	0 (0)	0 (0)
Frequently	5 (83)	9 (100)	0 (00	5 (83)	9 (100)	1 (100)
Research						
Not at all	0 (0)	1 (11)	0 (0)	0 (0)	1 (11)	0 (0)
Occasionally	2 (33)	5 (56)	0 (0)	3 (50)	4 (44)	1 (100)
Frequently	4 (68)	3 (33)	1 (100)	3 (50)	4 (44)	0 (0)
Education						
Not at all	0 (0)	0 (0)	0 (0)	0 (0)	0 (0)	0 (0)
Occasionally	3 (50)	1 (11)	0 (0)	2 (33)	0 (0)	0 (0)
Frequently	3 (50)	8 (89)	1 (100)	4 (67)	9 (100)	1 (100)

**Table 2:** **CNCs perceptions of components of the domain ‘Clinical Services Planning and Management’ (n = 16)**

**Grade**	**Functions**	**Grade 1**	**Grade 2**	**Grade 3**
		***n*** **= 6 (%)**	***n*** **= 9 (%)**	***n*** **= 1 (%)**
Grade 1	Identifies future issues and new directions for the service.	5 (83)	7 (78)	1 (100)
Grade 1	Contributes to formal service and strategic planning processes within the organisation.	5 (83)	8 (89)	1 (100)
Grade 1	Plans, implements and evaluates annual plan for nurse consultancy service.	3 (50)	2 (22)	0 (0)
Grade 2	Provides ongoing comprehensive analyses of current practice and the impact of new directions on the clinical specialty service.	5 (83)	5 (56)	0 (0)
Grade 2	Initiates, develops, implements and evaluates strategic changes for the clinical speciality/service.	3 (50)	5 (56)	1 (100)
Grade 3	Undertakes primary responsibility for preparation, implementation and evaluation of annual plan for the clinical service, e.g. Multidisciplinary business plan.	2 (33)	2 (22)	0 (0)
Grade 3	Manages complex projects relating to significant practice change for the organisation.	3 (50)	3 (33)	0 (0)
	Not applicable	0 (0)	0 (0)	0 (0)

**Table 3:** **CNCs perceptions of components of the domain ‘Clinical Services Planning and Consultancy’ (n = 16)**

**Grade**	**Functions**	**Grade 1**	**Grade 2**	**Grade 3**
		***n*** **= 6 (%)**	***n*** **= 9 (%)**	***n*** **= 1 (%)**
Grade 1	Provides an expert client-centred consultancy practice participating in direct patient care provision.	5 (83)	8 (89)	1 (100)
Grade 1	Provides education on complex clinical issues to clients and carers.	4 (67)	7 (78)	1 (100)
Grade 1	Identifies and adopts innovative clinical practice models e.g. implementation and evaluation of new treatments, technologies, and therapeutic techniques relating to CN/MC specialty.	3 (50)	8 (89)	1 (100)
Grade 1	Participates/collaborates in the design and conduct of quality improvement initiatives.	5 (83)	8 (89)	0 (0)
Grade 2	Provides a more complex and expansive clinical consultancy service within a mixed clinical environment and/or across a series of services.	4 (67)	8 (89)	1 (100)
Grade 2	Develops specialised education resources for patient/carer/community to be utilised by other health care professionals.	3 (50)	8 (89)	1 (100)
Grade 3	Provides a more complex and expansive clinical consultancy service within a mixed clinical environment and/or across multiple service groups	4 (67)	7 (78)	1 (100)
Grade 3	Undertakes primary responsibility for formalised ongoing clinical supervision processes for CN/MC peers	1 (17)	5 (56)	0 (0)
Grade 3	As an expert, conducts systematic review of clinical practice including, if required, for external organisations.	2 (33)	5 (56)	0 (0)
	Not applicable.	0 (0)	0 (0)	0 (0)

**Table 4:** **CNCs perceptions of components of the domain ‘Clinical Leadership’ (n = 16)**

**Grade**	**Functions**	**Grade 1**	**Grade 2**	**Grade 3**
		***n*** **= 6 (%)**	***n*** **= 9 (%)**	***n*** **= 1 (%)**
Grade 1	As an expert clinician, acts as a role model in the clinical setting.	5 (83)	9 (100)	1 (100)
Grade 1	Contributes to the development and management of clinical processes	5 (83)	9 (100)	1 (100)
Grade 1	Provides leadership in the ongoing review of clinical practice at facility or area health service, as required.	5 (83)	9 (100)	1 (100
Grade 2	Provides leadership in the ongoing review of clinical practice for a more complex service, i.e. a service provided at multiple sites or by multiple CNC/CMC’s across an area health service.	3 (50)	8 (89)	1 (100)
Grade 2	Participates in State and National working parties.	2 (33)	5 (56)	1 (100)
Grade 2	Assumes leadership roles, which promote broader advancement of clinical practice	2 (33)	4 (44)	1 (100)
Grade 3	Provides leadership in State, National and/or International nursing bodies and/or specialist clinical groups.	2 (33)	4 (44)	1 (100)
Grade 3	Initiates collaborative ventures with academic colleagues	2 (33)	3 (33)	0 (0)
	Not applicable.	1 (17)	0 (0)	0 (0)

**Table 5:** **CNCs perceptions of components of the domain ‘Research’ (n = 16)**

**Grade**	**Functions**	**Grade 1**	**Grade 2**	**Grade 3**
		***n*** **= 6 (%)**	***n*** **= 9 (%)**	***n*** **= 1 (%)**
Grade 1	Initiates, conducts and disseminates the findings of locally based research in specialty.	3 (50)	5 (56)	0 (0)
Grade 1	Participates as co-researcher in larger studies.	5 (83)	8 (89)	1 (100)
Grade 1	Manages research projects requiring clinical contribution from others.	3 (50)	2 (22)	1 (100)
Grade 2	Adapts and applies related scientific research to a clinical specialty	3 (50)	4 (44)	1 (100)
Grade 2	Initiates original research projects.	2 (33)	1 (11)	0 (0)
Grade 2	Disseminates own research results through specialist publications and presentations.	1 (17)	0 (0)	0 (0)
Grade 3	Acts as principal researcher in significant/large scale research studies	1 (17)	1 (11)	1 (100)
	Not applicable.	0 (0)	1 (11)	0 (0)

**Table 6:** **CNCs perceptions of components of the domain ‘Education’ (n = 16)**

**Grade**	**Functions**	**Grade 1**	**Grade 2**	**Grade 3**
		***n*** **= 6 (%)**	***n*** **= 9 (%)**	***n*** **= 1 (%)**
Grade 1	Participates in formal and informal education programs.	4 (67)	9 (100)	1 (100)
Grade 1	Identifies clinical education needs.	5 (83)	9 (100)	1 (100)
Grade 1	Collaborates with others in the development and delivery of education programs.	5 (83)	8 (89)	1 (100)
Grade 2	Undertakes primary responsibility for the planning and implementation of specialist clinical education for the local health network.	2 (33)	8 (89)	1 (100)
Grade 2	Develops significant education resources for nurses and other health care professionals.	1 (17)	8 (89)	1 (100)
Grade 2	Participates in the development and delivery of postgraduate tertiary programs.	1 (17)	2 (22)	0 (0)
Grade 3	Provides significant contribution to the direction of clinical nursing education within the speciality.	2 (33)	3 (33)	0 (0)
	Not applicable.	0 (0)	0 (0)	0 (0)

### Data collection

The CNCs and CMCs were recruited through their email addresses on the health district intranet. They were also given information at their monthly meetings by a CNC working in the Centre for Nursing Research and Practice Development (CNRPD) at Nepean Blue Mountains Local Health District (NBMLHD). They were sent an invitation including an information sheet and told that completion of the online survey was considered consent and that all identifying features would be removed before analysis. The participants were given instructions on how to access the online survey in Qualtrics [15] and told they could also use the electronic copy attached to their email if they felt more comfortable and return it to the CNRNP.

### Data analysis

Data from Qualtrics were downloaded into SPSS [14] and responses tallied. Descriptive statistics were calculated including totals, percentages, means and standard deviations and these are displayed in tables.

### Ethical considerations

Ethics approval was sought and given by the relevant University and Local Health District Human Research Ethics Committees. Data were collated and no identifying information was collected.

## Results

### Respondents

Sixteen (29.1%) of the population of CNCs/CMCs returned completed surveys. There were three eligible CMCs working in the health district and none of these answered the survey. Table 7 displays the demographic information of the respondents. There were six Grade 1 (37.5%), nine Grade 2 (56%), and one Grade 3 (6%) CNCs in the respondent group. No specific demographic details of the person who was the Grade 3 will be given to protect their identity. Of the 13 CNCs who provided demographic information, 10 were female (76.97%) and 23.1% (3) were male. Mean age for Grade 1 CNCs was 50.67 (SD = 8.02) and Grade 2 was 50.78 (SD = 7.98). Mean number of years working as a CNC was 6.5 (SD = 4.32) and 4.44 (SD = 4.13) respectively. The majority of the CNCs came from a less senior clinical position (12, 92.3%) to the role. Only six had a Master’s degree with most having a certificate in their specialisation. All the CNCs reported to a nurse as their line manager who they met with at least monthly. All but two had a written job description. Six CNCs (46.2%) had clerical assistance to take telephone massages, make appointments and do minimal typing and data entry.

**Table 7:** **Demographic details of participants (** ***n*** **= 16)**

**CNC Characteristics**	**Grade 1**	**Grade 2**
	**(** ***n*** **= 6)**	**(** ***n*** **= 9)**
Gender: Female	6	6
Male	0	3
Age (years)	39 – 62	35 – 60
	(M = 50.67; SD = 8.02)	(M = 50.78; SD = 7.98)
Number of years in speciality	1 – 32	1 – 36
	(M = 13.83; SD = 10.11)	(M = 15.89; SD = 10.56)
Number of years in current role	1 – 12	1 – 11
	(M = 6.5; SD = 4.32)	(M = 4.44; SD = 4.13)
*How did you get the job?*		
Transferred from other CNC position	1	1
Transferred from non CNC position	4	8
*Highest education*		
Masters	1	4
Bachelors	2	
Graduate certificate	2	5
Nil post registration education	1	

### Domains of the CNC role

Each of the five domains of practice was explored in the survey and the findings are depicted in tabulated form and discussed below. Additionally, each respondent was asked to provide an overall descriptor of their role using the findings of Fry et al. [2]. From the responses, six (37.5%) saw themselves as sole practitioners, six (37.5%) perceived themselves as team coordinators and four (25%) saw themselves as clinical coordinators. None of the respondents nominated themselves as a clinical leader.

#### Clinical service and management planning domain

Over 87.5% of the nurses perceived that clinical service and management is a frequent part of their role. However, only nine (56.3%) worked in this domain frequently and the rest occasionally. There was little variation across grades. In Table 2 it can be seen that all functions of the domain were seen as part of their role with the main functions being: *1. Identifies future issues and new directions for the service, 2. Contributes to formal service and strategically plans processes within the organisation and 3. Providing ongoing comprehensive analyses of current practice and the impact of new directions on the clinical specialty service* as the most common*.* This last component was not enacted as much by the Grade 2 s. While this may be the case, when Grades 1 and 2 CNCs were asked to estimate how many average hours a month were spent in this domain they were similar, with an average of 16.3 hours. The Grade 3 CNC spent eight hours per month enacting this domain.

#### Clinical services planning and consultancy

The majority of the CNCs (14, 87.5%) perceived that clinical service and consultancy should be a frequent part of their role and this was confirmed by 13 (81.3%) working in this domain frequently and the rest occasionally. There was little variation across grades (Table 1). In Table 3 it is shown that all functions of the domain were seen as part of the role with the main ones being: *1. provides an expert client-centred consultancy practice participating in direct patient care provision and 2. Participates/collaborates in the design and conduct of quality improvement initiatives.* Interesting the majority of Grade 2 and Grade 3 s included the following as components*: 1. Identifies and adopts innovative clinical practice models e.g. implementation and evaluation of new treatments, technologies, and therapeutic techniques relating to CN/MC specialty, 2. participates/collaborates in the design and conduct of quality improvement initiatives, and 3. provides a more complex and expansive clinical consultancy service within a mixed clinical environment and/or across a series of services.* Very few of the CNCs (7, 43.7%) did systematic reviews of clinical practice issues. When asked to estimate how many hours a month they engaged in these activities, the average for the Grades 1 and 2 were similar at 52.3 hours per month.

#### Clinical leadership

Most of the CNCs (15, 93.8%) perceived that clinical leadership should be a part of their role with 14 (87.5%) working in this domain frequently and the rest occasionally (Table 1). There was little variation across the grades. In Table 4 it can be seen that all functions of the domain were seen as part of the role with the most common functions being: *1. As an expert clinician, acts as a role model in the clinical setting, 2. Contributes to the development and management of clinical processes and 3. Provides leadership in the ongoing review of clinical practice at facility or area health service, as required.* There was little variation across grades with the other components not seen as part of the role by many CNCs. The average hours per month working in this domain were 32.6 with little variation across grades.

#### Research

Only seven CNCs (43.7%) perceived that research should be a frequent part of their role and this was confirmed by the fact that only eight (50%) did research frequently with one not doing it at all and the rest doing it occasionally Table 1. As indicated in Table 5 the most common function of the domain undertaken by the CNCs was *participates as co-researcher in larger studies* with the Grade 3 CNC acting *as a principal researcher* and was *managing projects and applying research to practice*. When asked how many hours a month they did this function the average was 27.6 hours with the Grade 3 CNC doing 35 hours.

#### Education

Fourteen (87.5%) of the CNCs saw that education should be a frequent part of their role with 12 (75%) stating that it was frequently part of their role. As displayed in Table 6 the most common functions perceived to be part of their role were: *1. Identifies clinical education needs and 2. Collaborates with others in the development and delivery of education programs*. On an average, this domain was part of the CNC role for 27.56 hours per month.

## Discussion

In this current study, the situation for the CNCs has not differed from the previous study in 2003 [6]. These findings support the assertion that the CNC’s are well-informed about the various responsibilities, domains and functions of their role, as stipulated by the NSW Award. The CNCs enacted the following domains in descending order over the three grades in an estimate of hours per month: clinical service and consultancy, clinical leadership, education clinical service planning and management and research. These findings align with those of Roche, Duffield, Wise [16] who also found clinical service and consultancy was the topmost domain within which the CNC practised.

The estimates of hours working in the specific domains, however, only show a trend as they do not represent a total of 37.5 hours per week that the CNCs work. As with other work on CNC’s [1] it may be construed that these nurses were engaged in local activities that are not easily covered by the existing domains and functions as outlined by the award. However, the award enables flexibility so that the role can be individually, locally and contextually constructed. Roche et al. [16] found that the CNC engaged in direct patient care and this may also be the case for these CNCs. These CNC’s found it difficult to retrospectively estimate the number of hours worked in the last month. Accurate data on the number of hours CNCs engage in various activities could only be collected using a time and motion study.

Despite none of the CNCs nominating themselves as clinical leaders, the CNCs in 2013 are acting in an expanded clinical leadership role which was not evident in the prior research of this group [3]. This may be demand driven or because there are more university programs in clinical leadership in this region of Sydney. Education is less evident in the enactment of roles and functions but this may be because in each of their specialities in the health district in which the CNCs work there are clinical nurse educators who fulfil this role.

Overall, the CNCs work over the five domains with the least emphasis on research, however the CNCs are initiating more research and subsequent publications compared to the earlier work by O’Baugh, Wilkes, Vaughan [3] and three CNC’s were principal investigators. Research is important in the health district and management supports it by providing secondments to the district’s nursing research unit and by providing funds for nurses’ research initiatives. Additionally, more of the CNCs have post graduate education which includes research methodologies. Overall CNCs need education and support to engage in research activities.

## Conclusion

This study is limited by the number of CNCs participating in the study. A further limitation is the use of a retrospective survey tool. Nevertheless, it has shown that although many of the nurses have been in their role for a long time, they still see themselves as experts who manage and consult. While they do not perceive themselves as clinical leaders, they perceive this domain of their role as very important.

Across this health district, there is no organisational consensus of the scope of practice of the CNC and it appears to be individually and contextually constructed. However, the Award clearly outlines the domains and functions within which these experts should practice. Supervisors and institutional leaders need to support and orientate nurses in these roles and accept that these nurses make contributions to the services that do not easily fit into the NSW Award [5]. The domains described in Fry et al. [2] may give a cleaner classification of roles and functions of the CNC, however each CNC enacts their role differently and their major focus continues to be enhancing patient care.

## Abbreviations

CNC: Clinical nurse consultant
CMC: Clinical midwife consultant

## References

[1] Baldwin R, Duffield CM, Fry M, Roche M, Stasa H, Solman A (2012). The role and functions of Clinical Nurse Consultants, an Australian advanced practice role: A descriptive exploratory cohort study. Int J Nurs Stud.

[2] Fry M, Duffield C, Baldwin R, Roche M, Stasa H, Solman A (2012). Development of a tool to describe the role of the clinical nurse consultant in Australia. J Clin Nurs.

[3] O’Baugh J, Wilkes LM, Vaughan K, O’Donohue R (2007). The role and scope of the clinical nurse consultant in Wentworth area health service, New South Wales, Australia. J Nurs Manag.

[4] Vaughan K, O’Baugh J, Wilkes LM, O’Donohue R (2005). The role and scope of the clinical nurse consultant in Wentworth area health service: a qualitative study. Collegian.

[5] NSW Health. Clinical Nurse Consultants- Domains and functions. Information bulletin. Workplace Relations and Management Branch. City; North Sydney 2011. Document number IB2011_024. http://www0.health.nsw.gov.au/policies/ib/2011/pdf/IB2011_024.pdf

[6] Donald F, Bryant-Lukosius D, Martin-Misener R, Kaasalainen S, Kilpatrick K, Carter N (2010). Clinical nurse specialists and nurse practitioners: title confusion and lack of role clarity. Can J Nurs Leadersh.

[7] Aranda K, Jones A (2008). Exploring new advanced practice roles in community nursing: a critique. Nurs Inq.

[8] Asbridge J (2006). New roles, new titles and the role of regulation. J Perioper Pract.

[9] Bloomer MJ, Cross WM (2011). An exploration of the role and scope of the clinical nurse consultant (CNC) in a metropolitan health service. Collegian: J Royal Coll Nurs Austr.

[10] Duffield CM, Gardner G, Chang AM, Fry M, Stasa H (2011). National regulation in Australia: A time for standardisation in roles and titles. Collegian: J Royal Coll Nurs Austr.

[11] Chang AM, Gardner GE, Duffield C, Ramis M-A (2010). A Delphi study to validate an advanced practice nursing tool. J Adv Nurs.

[12] Chang AM, Gardner GE, Duffield C, Ramis M-A (2012). Advanced practice nursing role development: factor analysis of a modified role delineation tool. J Adv Nurs.

[13] Leung S-O (2011). A comparison of psychometric properties and normality in 4-, 5-, 6-, and 11-point likert scales. J Soc Serv Res.

[14] IBM Corp. IBM SPSS Statistics for Windows. Version 22.0 City: New York. IBM Corp.; Released 2013. http://www-01.ibm.com/support/docview.wss?uid=swg21476197

[15] Qualtrics LLC. Qualtrics. Version: 6months, 2014. City: Provo, UT. Released 2005. http://www.qualtrics.com/

[16] Roche M, Duffield C, Wise S, Baldwin R, Fry M, Solman A (2013). Domains of practice and advanced practice nursing in Australia. Nurs Health Sci.

